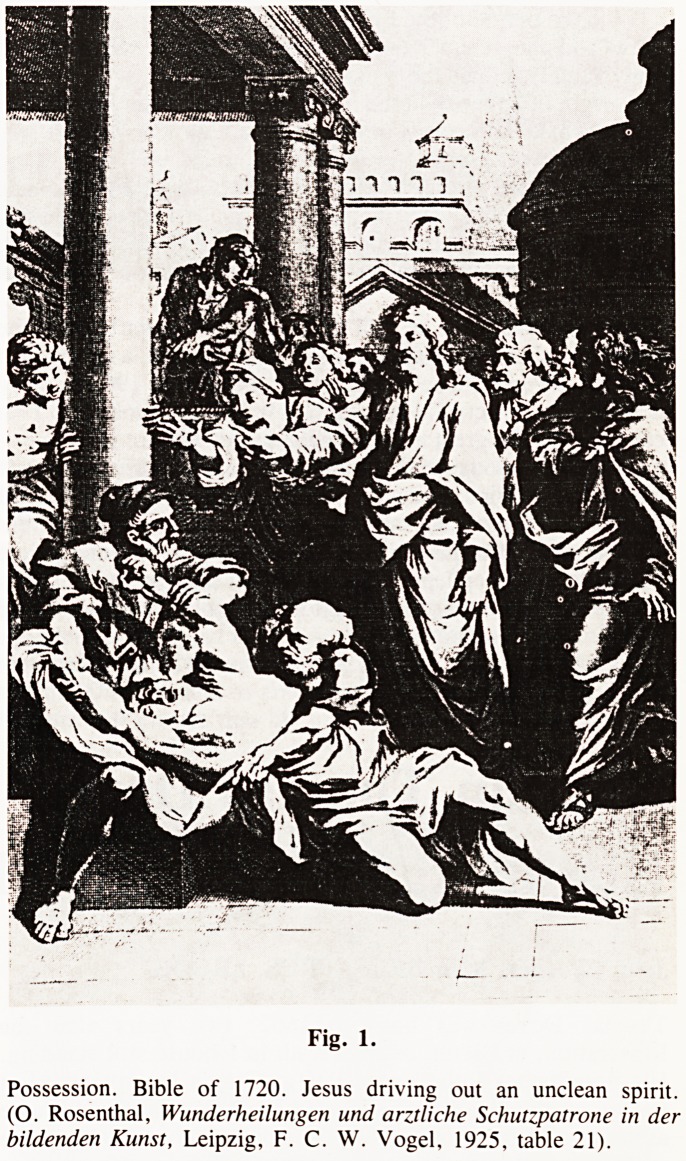# Epilepsy, Brain and Mind

**Published:** 1991-12

**Authors:** Jonathan Bird

**Affiliations:** BSc, MB ChB, MRCPsych


### Epilepsy — Brain and Mind

**Jonathan Bird, BSc, MB ChB, MRCPsych**

What is Epilepsy? In spite of the endless meetings of innumerable national and international committees we can still not arrive at a concerted opinion, the German neurologist Wolf wrote recently of epileptic seizure that "the term needs no explanation!" The British neurologist Reynolds follows this with the comment that "Epilepsy is a tendency to recurrent seizures" — which seems simple enough, but he then goes on to edit a whole book which still cannot arrive at a conclusion on "what is epilepsy?".

Epilepsy derives from the Greek word "epilambanein" meaning to seize, to be grabbed hold of, attacked. However, the symptoms were recognised and reported by the ancient Egyptians and Mesopotamians long before the Greeks. Dr. Reynolds has recently demonstrated a Babylonian stone tablet on which, in heiroglyphics, epilepsy was apparently described.

To the ancients of Mesopotamia and Egypt the disorder which we now call Epilepsy was considered a Sacred Disease in the sense of an invasion by the Gods — and in the struggle between magic and science, between dogma and reason in medicine, Epilepsy has held a key role ever since.

The ancients described seizures as an "antasubbu", relating it to the hand of sin and the God of the Moon — perhaps a tendency to nocturnal occurrence influenced this view.

However Hippocrates, or one of those early writers of about 400BC whom, we lump together under the name Hippocrates, wrote that it was neither sacred nor divine but that its cause lay in the brain, was hereditary and was due to an overflowing of phlegm. He also called it "The Great Disease", which has come down to us as "Grand Mal". He also took the enlightened view that the attacks were the epilepsy and there was no other necessary underlying illness.

In spite of these rather modern views, the Romans still regarded seizures as the work of demons — at least the Roman in the street did. Seizures were regarded as bad omens at any meeting, in spite, of course, of the reports that Julius Caesar suffered from them. They regarded epilepsy with particular horror and disgust, feeling that it was an "awesome" disease, evoking fear and the "sacred" really implied seized by demons.

The Roman physician Arateus described seizures well, clinically, as a convulsion of the whole body together with an impairment of the "leading functions" of conscious thought and response. He also described epilepsy as "a disease of various shapes and horrible".

Galen's view of epilepsy concurred with Arateus, and he considered that it was due to a thick humour gathering in the cerebral ventricles and blocking the passage of psychic phenomena — hence the origins of the nerves shake to push away what is distressing them, resulting in the convulsions. A patient of Galens' gave the first reported description of an "aura" as being "like a cold breeze climbing up" his body.

Seneca also described auras saying "it is useful to know one's disease and to suppress its powers before they spread". Many of the early physicians described patients who could prevent their seizures spreading by some mental activity.

During the Dark Ages, whilst most Doctors stuck to the Galenical view of seizures, the general public continued still to see epilepsy as the result of demon possession — especially based on Medieval Christian views. The famous descriptions of Jesus casting out a dumb spirit for instance.

At this time also there was great confusion about the overlap with what we would now term mental illness, "lunacy" was regarded as the same thing and all due to the same cause — the effect of the moon and evil spirits related to it.

Dante, in the Inferno, also describes a seizure as due to demons.

**Fig. 1.**

Possession. Bible of 1720. Jesus driving out an unclean spirit. (O. Rosenthal, *Wunderheilungen und arztliche Schutzpatrone in der bildenden Kunst*, Leipzig, F. C. W. Vogel, 1925, table 21).

Since epilepsy was due to unclean spirits, it was also regarded as infectious, the breath of a person with epilepsy was regarded as particularly dangerous. And so people with epilepsy were separated from the faithful and often refused the Eucharist.

With the coming of less dogmatic views and the possibility of debate allowed by the Renaissance, discussions sprung up as to whether epilepsy was due to possession or not. Attempts to classify the clinical symptomatology were made. In general the Galenial view that a convulsion with loss of senses was epilepsy of natural causes was upheld. Even the witch hunters manual, Malleus Maleficarium, admitted that natural epilepsy could be differentiated from witchcraft — though it was difficult to tell — and in general probably better to err on the side of caution and persecute them anyway. Further confusion occurred because of the observation that some people with epilepsy entered into what were termed "Ecstasies" or prophetic trances — indeed Mohammed was reported to have done so.

A delightful description of a confusing case and the vacillations of one Doctor Coboldus is given by Casaubon — an Oxford divine writing in 1655.

Whilst in general this was an era of careful observations, there was a proliferation of theories of causation — in particular Paracelsus who took a complex view based on alchemy and the balance of elements within man. He saw epilepsy as equivalent in man, the microcosm, to a thunderstorm in the macrocosm and both due to the element of fire getting out of hand. A further view was based on the observation that sudden fright might result in a seizure and that epilepsy and hysteria overlapped and were the result of the wandering uterus becoming dislodged.

With the period of the Enlightenment came a more scientific view and the renunciation of witchcraft as a cause. Willis convincingly refuted the wandering uterus as a cause and placed the origin of epilepsy in the brain. He had a variety of clinical and mechanical theories of causation but basically felt it was because the brain was of a weak constitution and allowed the turbulent animal spirits lying in the centre of the brain to explode upwards. The physician Cheyne took a rather mechanical view that epilepsy was due to disturbance of the elastic fibres (the nerves) which were rather like the strings of a musical instrument.

It was at this stage as well that witchcraft crept back in the guise of sex, and epilepsy blamed on masturbation, a view which became so established that even the great 19th century neurologist Gowers did not refute it in his text book. The 19th century was the great and energetic era of asylum building — with the separation of epileptics into colonies or at least, separate wards. In the Salpetrière and Bicêtre in Paris Pinel and Esquirol were able to begin studying great numbers of epilepsy sufferers and to define terms as well as study the psychological manifestations of epilepsy. Pinel classified epilepsy as a neurosis of cerebral functions. Morel and Falret looked for underlying personality characteristics. Morel wrote that he had "found the epileptic within epilepsy" and studied the life and character of his patients, describing "larval epilepsy" — as part of his general "degeneracy theories" — a view that hereditary influences could lead to an inevitable deterioration from generation to generation — through criminality, mental disease, epilepsy, dementia and idiocy. Falret was particularly interested in epileptic insanity and automatisms which he regarded as imperfect fits and called "petit mal intellectual".

In the U.K. there was a rather different emphasis, much less of a psychiatric one and more of a neurological one. Neurology was fighting to develop as a separate discipline, with the setting up of the National Hospital for the paralysed and epileptic in 1857 by Marshall-Hall and Brown-Sequard. The neurologists Russell Reynolds, Todd, Bright and others began carefully to describe epilepsy as it presented to them outside the asylums.

Russell-Reynolds found that very few patients with epilepsy were mentally deficient or insane (especially in his private practice). He regarded idiopathic generalised epilepsy as a disease in its own right but tended to exclude partial seizures. In 1860 the first specific anticonvulsants — the Bromides — were introduced.

It was also in the 1860's that the great Hughlings-Jackson widened the concept of epilepsy to include what we would now call Complex Partial Epilepsy — partial fits other than the generalised convulsions. The modern understanding of epilepsy starts with Huglings-Jackson.

## Figures and Tables

**Fig. 1. f1:**